# The effects of agmatine treatment on cisplatin-induced hepatotoxicity: an experimental rat study

**DOI:** 10.1590/1516-3180.2025.3392.02032026

**Published:** 2026-06-01

**Authors:** Murat Yeniceri, Mustafa Can Senoymak, Süleyman Bas, Musa Salmanoglu, Alpaslan Tanoglu

**Affiliations:** I Physician, Department of Rheumatology, University of Health Sciences, Kartal Dr. Lutfi Kirdar City Hospital, Istanbul, Türkiye.; II Physician, Department of Endocrinology, University of Health Sciences, Sultan 2. Abdulhamid Han Training and Research Hospital, Istanbul, Türkiye.; III Assistant Professor, Department of Internal Medicine, University of Health Sciences, Sancaktepe Sehit Prof. Dr. Ilhan Varank Training and Research Hospital, Istanbul, Türkiye.; IV Assistant Professor, Department of Internal Medicine, University of Health Sciences, Sultan 2. Abdulhamid Han Training and Research Hospital, Istanbul, Türkiye.; V Professor, Division of Gastroenterology, Department of Internal Medicine, Bahçeşehir University, Istanbul, Türkiye.

**Keywords:** Agmatine, Cisplatin, Chemical and drug-induced liver injury, Oxidative stress, Rats, Agmatine treatment, Cisplatin toxicity, Oxidative liver damage, Inflammation, Experimental rat study, Hepatoprotection

## Abstract

**BACKGROUND:**

Cisplatin is a widely used chemotherapeutic agent whose clinical utility is limited by dose-dependent hepatotoxicity, primarily driven by oxidative stress and inflammation. Agmatine, an endogenous polyamine, has demonstrated antioxidant and anti-inflammatory properties in various models; however, its hepatoprotective effects in cisplatin-induced liver injury remain underexplored.

**METHODS:**

This experimental study investigated the protective effects of agmatine against cisplatin-induced hepatotoxicity in 28 male Sprague Dawley rats. The animals were randomized into four groups: Sham, Cisplatin, Cisplatin + Agmatine (20 g/kg/day, orally), and Placebo. Biochemical markers, including ALT, AST, MDA (malondialdehyde), SOD (superoxide dismutase), GPx (glutathione peroxidase), TNF-α, IL-1β, and TGF-β, were assessed using ELISA (enzyme-linked immunosorbent assay). Histopathological evaluation was performed to assess liver architecture and injury severity.

**RESULTS:**

Cisplatin administration significantly increased serum levels of ALT, AST, MDA, and proinflammatory cytokines (IL-1β, TNF-α, and TGF-β), while reducing antioxidant enzyme activities (SOD and GPx) (p < 0.01). Agmatine co-administration significantly reversed these biochemical alterations and ameliorated histopathological damage, as evidenced by reduced inflammatory infiltration, sinusoidal dilatation, and hepatocellular degeneration.

**CONCLUSION:**

Agmatine demonstrates significant hepatoprotective effects against cisplatin-induced liver injury by attenuating oxidative stress and inflammatory responses. These findings suggest its potential role as an adjunctive agent to improve the hepatic safety profile of cisplatin chemotherapy.

## INTRODUCTION

 Cisplatin (cis-diamminedichloroplatinum) is a platinum-based chemotherapeutic agent that is extensively used in the treatment of various malignancies because of its potent antineoplastic activity.^1^ However, its clinical application is often hampered by severe adverse effects, among which hepatotoxicity holds significant clinical relevance. This toxicity is characterized by elevated serum liver enzyme levels and histopathological changes that compromise hepatic function and may adversely affect therapeutic outcomes.^2^

 The hepatotoxic effects of cisplatin are primarily mediated by oxidative stress, resulting in excessive production of reactive oxygen species (ROS), lipid peroxidation, and protein peroxidation, as well as inhibition of endogenous antioxidant defense mechanisms.^3^ These events lead to hepatocellular injury, inflammation, and apoptosis. Therefore, there is an ongoing need for adjunctive agents that can mitigate these adverse hepatic effects without diminishing the chemotherapeutic efficacy of cisplatin. 

 Agmatine, an endogenous polyamine derived from the decarboxylation of L-arginine by arginine decarboxylase, exhibits multiple pharmacological properties, including modulation of nitric oxide synthesis, interaction with imidazoline and α2-adrenergic receptors, and antagonism of NMDA receptors.^4-6^ Its antioxidant, anti-inflammatory, and anti-apoptotic effects have been demonstrated in various organ systems; however, its hepatoprotective potential, particularly in the context of cisplatin-induced toxicity, remains inadequately explored.^7,8^

 This study was designed to evaluate the protective effects of agmatine against cisplatin-induced hepatotoxicity in an experimental rat model and to elucidate its potential therapeutic role by employing comprehensive biochemical assays and histopathological analyses. 

## MATERIALS AND METHODS

### Study design

 This study was conducted at the Experimental Animal Laboratory, University of Health Sciences, Istanbul, Türkiye, following approval from the Institutional Animal Ethics Committee (Protocol No 2020-05-06) and in accordance with ARRIVE 3.0 guidelines. 

 A total of 28 male Sprague-Dawley rats, with body weights ranging from 250 to 350 g, were included in the study. The animals were housed under controlled laboratory conditions (12-h light/dark cycle, ambient temperature of approximately 24^°^C) and had ad libitum access to food and water. Food intake was restricted for 12-h prior to drug administration to standardize the baseline metabolic state. 

 The experimental timeline included agmatine administration, cisplatin injection, and euthanasia, which were performed in accordance with the predefined study protocol. 

 The rats were randomly divided into four experimental groups (n = 7 per group) as follows: 

**Group 1** – Sham: no intervention.

**Group 2** – Cisplatin: single intraperitoneal (i.p.) injection of cisplatin (12 mg/kg) on Day 5; animals were euthanized on Day 8.

**Group 3** – Cisplatin + Agmatine: oral agmatine (20 mg/kg/day) for 7 days; cisplatin administered on Day 5; euthanasia on Day 8.

**Group 4** Placebo: oral saline (1 mL, 0.9% NaCl) for 7 days; cisplatin (12 mg/kg) administered on Day 5; euthanasia on Day 8.

### Biochemical and histopathological evaluation

 On Day 8 of the study, all animals were anesthetized through an intraperitoneal injection of xylazine (10 mg/kg) and ketamine (75 mg/kg). Following midline laparotomy, blood samples were taken from the inferior vena cava. After excision of the liver tissue, the animals were euthanized humanely. 

 Serum biomarkers were evaluated to reflect systemic oxidative stress and inflammatory responses, which are widely used indicators in experimental hepatotoxicity studies. 

 Blood samples were immediately centrifuged at 3000 rpm for 10 min, and the resulting serum was stored at −70^°^C until subsequent analysis. The following biochemical parameters were quantified using rat-specific solid-phase enzyme-linked immunosorbent assay (ELISA) kits (BT Lab, China) in accordance with the manufacturer’s protocols: alanine aminotransferase (ALT), aspartate aminotransferase (AST), tumor necrosis factor-alpha (TNF-α), transforming growth factor-beta (TGF-β), malondialdehyde (MDA), superoxide dismutase (SOD), glutathione peroxidase (GPx), and interleukin-1 β (IL-1β). 

 For histopathological evaluation, liver specimens were fixed in 10% neutral-buffered formalin, embedded in paraffin, and cut into sections. The tissue sections were stained with hematoxylin and eosin (H&E) and examined under a light microscope (Nikon Eclipse E600). Liver injury was assessed semi-quantitatively based on four histological parameters: inflammatory cell infiltration, sinusoidal dilatation, hepatocyte damage, and vascular congestion. Each parameter was scored according to the following scale: The severity of liver tissue injury was graded as follows: score − (no histopathological damage), score + (mild damage affecting < 25% of the liver tissue), score ++ (moderate damage affecting 25–50% of the tissue), and score +++ (severe damage affecting > 50% of the tissue). All histopathological evaluations were conducted by an experienced pathologist blinded to the experimental groups to minimize observer bias. 

### Statistical Analyses

 All statistical analyses were performed using SPSS version 25.0 (IBM Corp., Armonk, New York). Descriptive statistics, including means and standard deviations (SDs), were calculated for all variables. Normality of the data distribution was assessed using the Shapiro–Wilk test and visual inspection of histograms and Q–Q plots. For data exhibiting a normal distribution, comparisons between two groups were conducted using an independent samples t-test, whereas one-way analysis of variance (ANOVA) was used for comparisons involving more than two groups. Post hoc multiple comparisons were performed using the Bonferroni test. Homogeneity of variances was assessed prior to parametric analyses. Statistical significance was set at p < 0.05, and all results were reported with a 95% confidence interval (95% CI). 

## RESULTS

 The serum levels of ALT, AST, GPx, IL-1β, SOD, TGF-β, MDA, and TNF-α measured in all experimental groups are summarized in **Table 1**. 

**Table 1:** Biochemical parameters of study groups

**Parameter**	**Group 1 (SaM)**	**Group 2 (Hepatotoxic)**	**Group 3 (Hep + Treatment)**	**Group 4 (Placebo)**	**p value**
ALT (U/L)	33.4 ± 2.3^*^	60.87 ± 4.87^*^	48.25 ± 3.33^*^	58.14 ± 4.72^*^	< 0.001^a^
AST (U/L)	37.69 ± 3.57^*^	59.94 ± 3.93^*^	48.04 ± 2.58^*^	57.02 ± 3.53^*^	< 0.001^a^
GPx (ng/mL)	17.93 ± 1.2^*^	14.53 ± 0.62^*^	16.76 ± 0.99^*^	14.66 ± 1.02^*^	< 0.001^a^
IL-1β (pg/mL)	501.44 ± 41.24^*^	636.69 ± 32.27^*^	550.74 ± 25.57^*^	612.88 ± 45.31^*^	< 0.001^a^
Superoxide dismutase (ng/L)	1.14 ± 0.1^*^	0.78 ± 0.04^*^	1 ± 0.06^*^	0.82 ± 0.06^*^	< 0.001^a^
TGF-β (ng/L)	117.07 ± 10.03^*^	140.08 ± 5.51^*^	128.7 ± 4.71^*^	136.51 ± 5.41^*^	< 0.001^a^
TNF-α (ng/mL)	49.05 ± 8.52^*^	67.65 ± 4.09^*^	50.97 ± 4.96^*^	62.88 ± 3.75^*^	< 0.001^a^
Malondialdehyde (nmol/mL)	0.78 ± 0.06^*^	1.28 ± 0.07^*^	0.96 ± 0.08^*^	1.27 ± 0.07^*^	< 0.001^a^

* p < 0.01

a Significantly different compared with the control group;

b Significantly different compared with the CP group; ALT, alanine aminotransferase; AST, aspartate aminotransferase; GPx, glutathione peroxidase; IL-1β, interleukin-1 beta; SOD, superoxide dismutase; TGF-β, transforming growth factor beta; TNF-α, tumor necrosis factor alpha; MDA, malondialdehyde.

**Figure 1:**
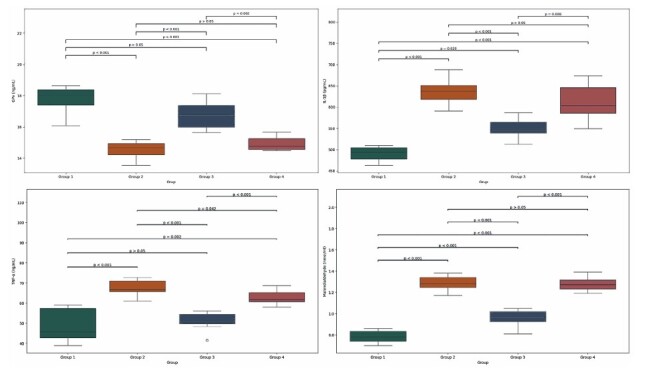
Comparison of (A) Gp-x, (B) IL-1β, (C) TNF-α, and (D) MDA levels among Group 1, Group 2, Group 3 and Group 4.

 Cisplatin administration (Group 2) resulted in a significant elevation in serum ALT, AST, IL-1β, TGF-β, TNF-α, and MDA levels compared to the Sham group (Group 1; p < 0.01). Conversely, cisplatin exposure caused a notable reduction in the levels of the antioxidant enzymes SOD and GPx. No statistically significant differences were observed between the Cisplatin-only group and the Placebo group (Group 4), indicating that oral saline administration did not modify the effects of cisplatin (**Figures 1** **and** **2**). 

Cisplatin administration significantly increased serum levels of ALT, AST, MDA, and proinflammatory cytokines (IL-1β, TNF-α, and TGF-β), while reducing antioxidant enzyme activities (SOD and GPx) (p < 0.01). Agmatine co-administration significantly reversed these biochemical alterations and ameliorated histopathological damage, as evidenced by reduced inflammatory infiltration, sinusoidal dilatation, and hepatocellular degeneration

**Figure 2:**
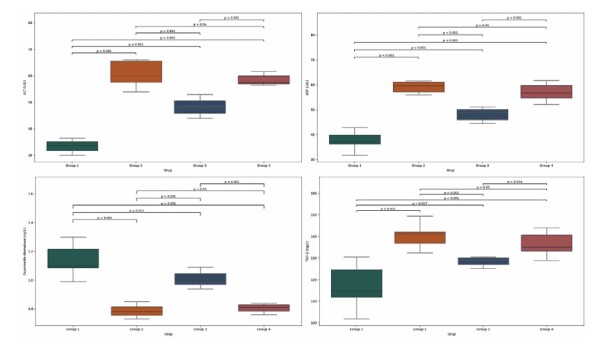
Comparison of (A) ALT, (B) AST, (C) SOD and (D) TGF-β levels among Group 1, Group 2, Group 3 and Group 4.

The Kruskal–Wallis test (nonparametric ANOVA) was used for all statistical comparisons. ALT and AST levels were significantly higher in Group 2 than in all other groups (p < 0.001). SOD activity was reduced in Groups 2 and 4 compared to Group 1 (p < 0.001). TGF-β levels were elevated in Group 2 (p < 0.001). ANOVA, analysis of variance; Group 1, sham group; Group 2, cisplatin group; Group 3, cisplatin + agmatine group; Group 4, control group; ALT, alanine aminotransferase; AST, aspartate aminotransferase; SOD, superoxide dismutase; TGF-β,transforming growth factor beta.

 In contrast, rats in the Cisplatin + Agmatine group (Group 3) exhibited significantly lower levels of ALT, AST, IL-1β, TGF-β, TNF-α, and MDA than those in the Cisplatin-only and Placebo groups (p < 0.01). 

 Additionally, GPx and SOD levels were significantly elevated in the agmatine-treated group, demonstrating the restoration of antioxidant defense mechanisms. 

 Histopathological examination corroborated the biochemical findings. In the sham group, liver tissues retained normal architecture with intact hepatocyte morphology. In contrast, the cisplatin group displayed severe hepatic damage, including hydropic degeneration of hepatocytes, sinusoidal dilatation, hyperemia, focal necrosis, and inflammatory cell infiltration. Similar pathological alterations were observed in the placebo group (**Table 2**). 

**Table 2:** Grading of the histopathological changes in the liver sections of rats. Scoring was performed as follows: none (−), mild (+), moderate (++) and severe (+++)

**Groups**	**Cell Infiltration**	**Dilation of Sinusoids**	**Hepatocyte Injury**	**Vascular Congestion**
Group 1. Sham	−	−	−	−
Group 2. Cisplatin	+++	+++	+++	+++
Group 3. Cisplatin + Agmatine	+	+	+	++
Group 4. Placebo	+++	+++	+++	+++

**Figure 3:**
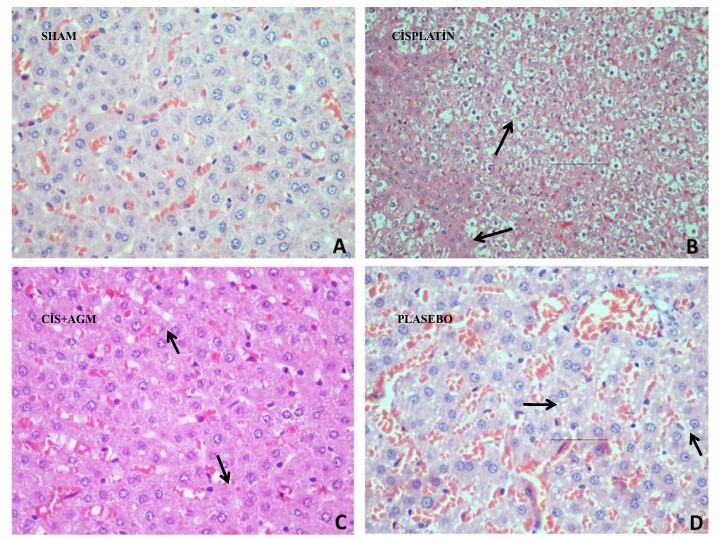
Histopathological evaluation of study groups.

 However, in the Cisplatin + Agmatine group, significant histological improvements were observed. Notably, the extent of hydropic degeneration, sinusoidal dilatation, and inflammatory infiltration was markedly reduced compared to that in the Cisplatinonly and Placebo groups (**Figure 3**). These results further support the hepatoprotective effect of agmatine in alleviating cisplatin-induced liver injury. 

Light microscopy images of the liver tissues stained with hematoxylin and eosin at × 100 magnification. (A) Sham group (Group 1) with normal liver tissue. (B) Cisplatin group (Group 2) with hepatocyte injury and cell infiltration. (C) Cisplatin + Agmatine group (Group 3) with reduced cell infiltration and mild injury. (D) Placebo group (Group 4) with sinusoidal dilatation.

## DISCUSSION

 To the best of our knowledge, this is the first experimental study to assess the protective potential of agmatine against cisplatininduced hepatotoxicity. These findings suggest that agmatine may serve as a promising adjunctive therapy to enhance the safety profile of cisplatin-based chemotherapy regimens. 

 Cisplatin-induced hepatotoxicity is primarily attributed to oxidative stress and inflammation.^9,10^ Consistent with previous studies, our findings show that cisplatin exposure led to elevated levels of MDA and proinflammatory cytokines (IL-1β, TNF-α, and TGF-β), along with a reduction in the activities of antioxidant enzymes, such as SOD and GPx.^11-13^ These alterations indicate enhanced lipid peroxidation and a compromised antioxidant defense system, ultimately contributing to hepatocellular apoptosis and structural deterioration. Histopathological analysis corroborated these biochemical findings, revealing substantial hepatocyte damage, sinusoidal dilatation, and inflammatory infiltration in cisplatin-treated rats. The biochemical findings were consistent with histopathological observations, supporting the protective effect of agmatine against hepatocellular injury. 

 The role of oxidative stress in cisplatin-induced hepatotoxicity has been well established. Previous studies have demonstrated that cisplatin administration increases ROS production, leading to lipid peroxidation and oxidative damage to cellular proteins and nucleic acids.^13-15^ This cascade of events activates inflammatory pathways, exacerbating tissue injury and impairing liver function. Our study is consistent with this mechanistic understanding, as evidenced by the elevated MDA levels and decreased SOD and GPx activities observed in the cisplatin-treated group. Furthermore, the increased levels of IL-1β, TNF-α, and TGF-β in the cisplatin group support the inflammatory response associated with liver injury. 

 Given the pathophysiology of cisplatin-induced liver injury, the use of agents with both antioxidant and anti-inflammatory properties has been proposed as a viable therapeutic strategy. Agmatine was selected in this study because of its well-documented ability to modulate oxidative stress and inflammation. In our experiment, agmatine significantly attenuated the expression of proinflammatory cytokines, decreased serum ALT and AST levels, and improved antioxidant enzyme profiles. These biochemical improvements were accompanied by a marked reduction in histopathological damage, underscoring agmatine’s dual protective mechanism. The reduction in ALT and AST levels, which are indicative of hepatocellular injury, further supports the notion that agmatine can mitigate liver damage. Additionally, the restoration of antioxidant enzyme activity, particularly SOD and GPx, suggests that agmatine’s hepatoprotective effects are, at least in part, mediated through the restoration of the hepatic antioxidant defense system. 

 Our findings are consistent with previous studies that have investigated the protective role of agmatine in hepatic and extrahepatic tissues. For instance, Ahmed et al. observed similar protective effects of agmatine in a model of valproic acid-induced liver injury, where agmatine reduced serum aminotransferase levels, attenuated proinflammatory cytokine levels, and improved histological parameters.^16^ Although their study focused on tissue SOD levels rather than serum levels, as in our study, the directionality of the response was consistent, reinforcing the broad therapeutic potential of agmatine in preventing various forms of hepatic injury. Moreover, Alharbi et al. demonstrated that agmatine significantly mitigated oxidative stress and inflammation in rat testicular tissue exposed to tacrolimus, a potent immunosuppressant.^17^ Similarly, Yeniceri et al.^18^ showed that agmatine treatment reduced inflammation and oxidative damage in pancreatic tissue, further highlighting its systemic anti-inflammatory and antioxidant properties. These findings collectively support agmatine’s broad therapeutic potential as an agent capable of attenuating drug-induced toxicities across multiple organ systems. 

 In addition to its hepatoprotective effects, agmatine has been explored in models of cisplatin-induced toxicity in other organ systems.^19-22^ Donertas et al.^19^ demonstrated that agmatine alleviated degeneration of the dorsal root ganglion and sciatic nerve in a rat model of cisplatin-induced peripheral neuropathy. This finding further supports the neuroprotective potential of agmatine. 

 Similarly, studies on cisplatin-induced nephrotoxicity have shown that agmatine improves renal function parameters and histopathological outcomes, highlighting its systemic protective capacity. These studies emphasize the multifaceted nature of agmatine’s protective effects and its potential as a therapeutic agent for mitigating cisplatin-induced toxicities in various organ systems.^10^

 This study has several limitations. First, the sample size was relatively limited, which may restrict the generalizability of the findings. Second, although biochemical and histopathological evaluations were comprehensively performed, detailed molecular analyses investigating the underlying mechanisms were not conducted. Future studies with larger sample sizes and molecular investigations are needed to further clarify the protective mechanisms of agmatine in cisplatin-induced hepatotoxicity. 

 In conclusion, agmatine effectively mitigates cisplatin-induced hepatotoxicity by attenuating oxidative stress and inflammatory cytokine responses while preserving hepatic morphology and function. These findings propose agmatine as a promising adjunctive agent in oncology to safeguard liver function during cisplatin chemotherapy. Further studies are warranted to explore its translational applicability and systemic protective profile in other cisplatin-affected organ systems. 

## Data Availability

Data supporting the findings of this study are available from the corresponding author, Murat Yeniceri, upon request.
